# Supplementary material for: Adjuvant Curdlan Contributes to Immunization against Cryptococcus gattii Infection in a Mouse Strain-Specific Manner
Source: Vaccines (Basel). 2022 Apr 15;10(4):620. doi: 10.3390/vaccines10040620 (PMC9030172; doi:10.3390/vaccines10040620)
Supplement: Supplementary file 1 [file vaccines-10-00620-s001.zip › vaccines-1630231-supplementary.pdf]

vaccines

Supplementary Material

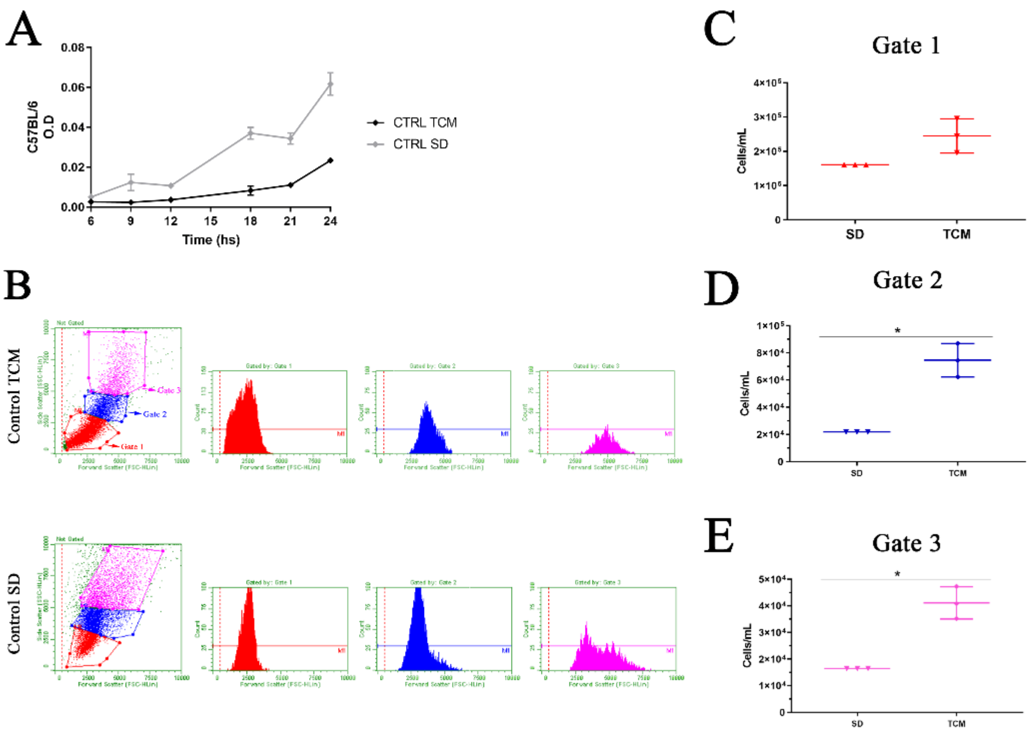

Figure S1. Protocol for the titanization of *C. gattii* using TCM. **(A)** *C. gattii* (5 × 10^4 yeast/mL) were cultivated in SD medium or TCM, and the growth curve was determined by measuring absorbance (optical density [OD]) at 540 nm (over time for 24 h). **(B–E)** *C. gattii* culture was inactivated by heating, and the cell suspension was divided into three populations with distinct sizes (as represented by gates 1, 2, and 3) based on the FSC-HLin parameter, using flow cytometry. **(C–E)** The concentration (cell/mL) of *C. gattii* was also determined by flow cytometry. Results are expressed as mean ± SD. * $p < 0.05$.

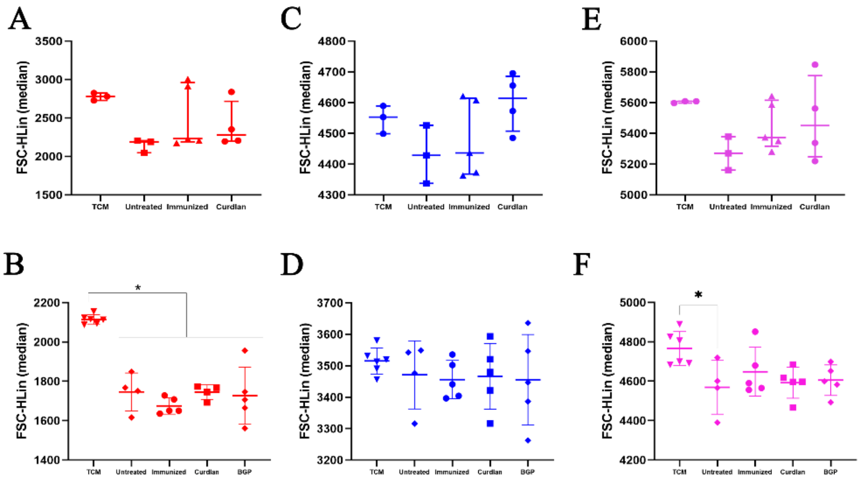

Figure S2. Validation of the method used to separate a cell suspension of *C. gattii* in three distinct populations based on cell size via flow cytometry. BALB/c and C57BL/6 mice received curdlan, PBS, or BGP before immunization with HK-*C. gattii*-thin. The mice were subsequently challenged with *C. gattii*. Serum was collected from mice after 14 days of infection and incubated with *C. gattii* cultured in TCM. After 24 h of incubation, *C. gattii* was analyzed by flow cytometry to quantify the cell concentration of *C. gattii* as described in Figures 3b–d and 12c–e. The separation of cell suspension in three distinct populations was performed using gates 1 (A-B, red), 2 (C-D, blue), and 3 (E-pink), as shown in Figures 3a and 12b. To demonstrate that the cell population gating was homogenous

across the groups, cell size was measured using the FSC-HLin parameter in flow cytometry. For each gate, the absence of a significant difference in the cell size between the untreated, immunized, curdlan, and/or BGP groups validates the separate homogeneous populations. Results are expressed as medians and interquartile ranges. * $p < 0.05$.

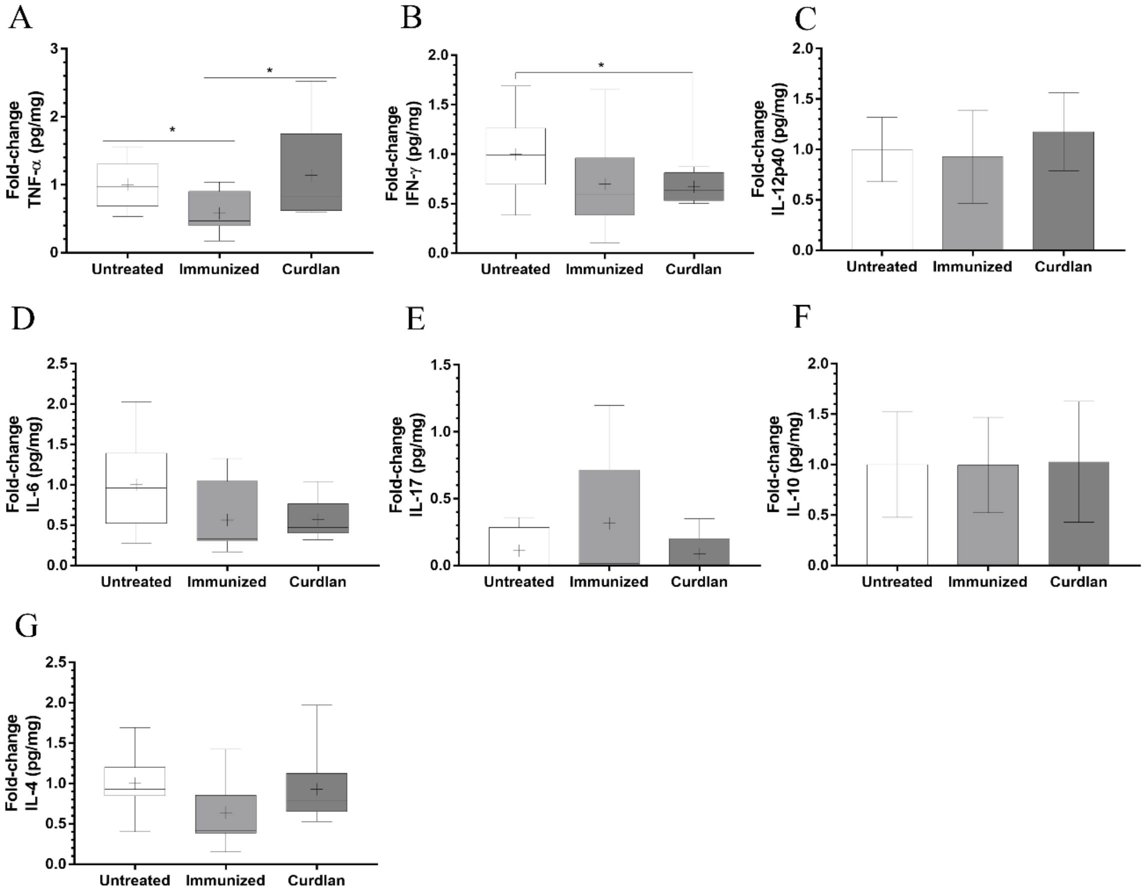

Figure S3. Measurement of cytokine levels in the lungs of BALB/c mice immunized against and challenged with *C. gattii*. After the administration of curdlan or PBS, BALB/c mice were immunized with HK-*C. gattii*-thin. On day 45 (14 d.p.i.), the mice were challenged with *C. gattii*. On day 59 (14 days post-infection), the lungs of these mice were harvested, homogenized, and the supernatants were used to measure the levels of **(A)** TNF-α, **(B)** IFN-γ, **(C)** IL-12p40, **(D)** IL-6, **(E)** IL-17, **(F)** IL-10, and **(G)** IL-4 via ELISA. Results are expressed as mean ± SD or as medians with interquartile ranges. * $p < 0.05$.

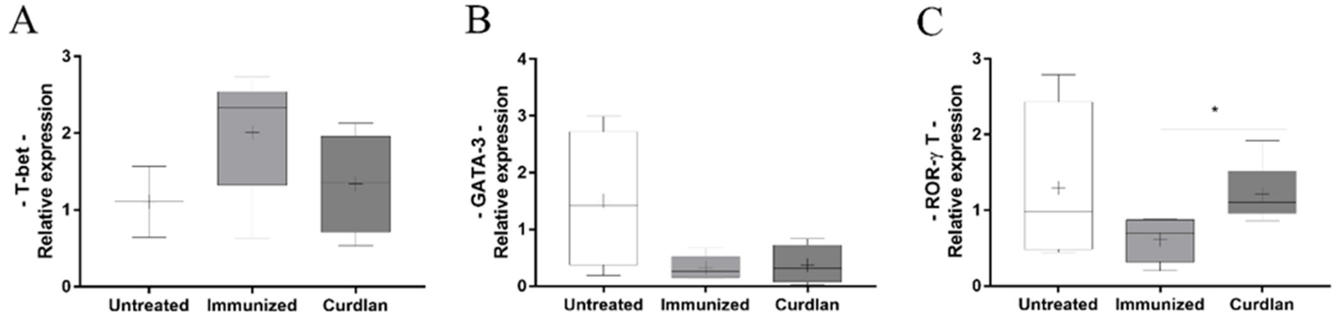

Figure S4. Quantification of the relative expression of transcription factors related to T helper cell differentiation in the lungs of C57BL/6 mice immunized against and challenged with *C. gattii*. After the administration of curdlan or PBS, C57BL/6 mice were immunized with HK-*C. gattii*-thin. On day 45 (14 d.p.i.), the mice were challenged with *C. gattii*. On day 59 (14 days post-infection), the lungs of these mice were harvested and homogenized in Trizol reagent to extract the total RNA. The cDNA was generated and used to measure the relative expression of levels of *T-bet* **(A)**, *GATA-3* **(B)**, and

ROR-γt **(C)** by qRT-qPCR. The values were normalized to *β-actin* expression. Results are expressed as mean ± SD or as medians with interquartile ranges. * $p < 0.05$.

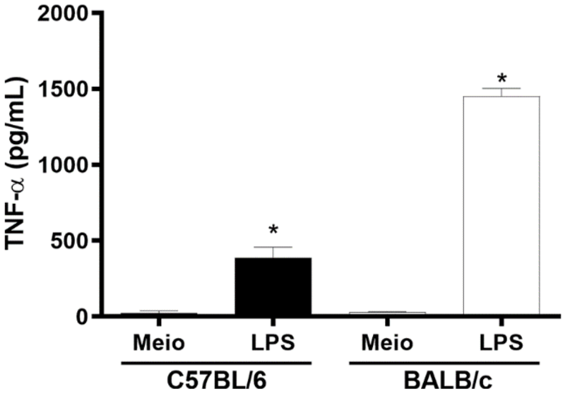

Figure S5. Quantification of TNF-α levels in the culture supernatant of adherent lung cells from C57BL/6 and BALB/c mice. Adherent lung cells (1×10^5 cell/mL) from C57BL/6 and BALB/c mice were incubated with medium (RPMI) or lipopolysaccharide (LPS; 100 ng/mL) for 24 h, and TNF-α was measured in the supernatant. Results are expressed as mean ± SD. * $p < 0.05$.
